# Revision of restrictive bariatric procedures in elderly patients: results at a 5-year follow-up

**DOI:** 10.1007/s13304-024-01888-2

**Published:** 2024-05-26

**Authors:** Adam Abu-Abeid, Noa Gosher, Mati Shnell, Sigal Fishman, Andrei Keidar, Guy Lahat, Shai Meron Eldar

**Affiliations:** 1 Division of General Surgery, Tel Aviv Sourasky Medical Center, Affiliated to Sackler Faculty of Medicine, Tel Aviv University, 6 Weizman Street, 64230906 Tel Aviv, Israel; 2 Division of General Surgery, Bariatric Unit, Tel Aviv Sourasky Medical Center, Affiliated to Sackler Faculty of Medicine, Tel Aviv University, Tel Aviv, Israel; 3 Department of Gastroenterology and Liver Diseases, Tel Aviv Sourasky Medical Center, Affiliated with Sackler School of Medicine, Tel Aviv University, Tel Aviv, Israel

**Keywords:** Metabolic and bariatric surgery, Revisional bariatric surgery, Elderly, Safety, Long-term weight loss

## Abstract

Revisional Bariatric Surgery (RBS) is increasing in popularity. Elderly patients (> 65 years old) are sometimes referred for RBS evaluation. The aim of this study is to evaluate outcomes of elderly patients undergoing RBS. A retrospective analysis of a cohort from a single–tertiary bariatric center. All elderly patients undergoing RBS after restrictive procedures between 2012 and 2022 were included. Thirty Nine patients undergoing RBS were included in the comparative analysis − 23 patients (57.5%) after adjustable gastric banding (s/p LAGB) and 16 patients (40%) after Sleeve Gastrectomy (s/p SG). The mean age and body mass index (BMI) of patients were comparable (67.2 ± 2.8 years and 38.3 ± 7.4, respectively). There was no difference in associated medical problems except reflux which was higher in s/p SG (68% vs. 13%; *p* < 0.001). The mean time interval between surgeries was 8.7 ± 5.1 years. The surgeries included One anastomosis gastric bypass (*n* = 22), SG (*n* = 8) and Roux-en-y gastric bypass (*n* = 9). Early major complication rates were comparable (4.3% and 12.5%; *p* = 0.36), and readmission rate was higher in patients s/p SG (*p* = 0.03). Ninety percent of patients were available to a follow-up of 59.8 months. The mean BMI and total weight loss was 29.2 and 20.3%, respectively with no difference between groups. The rate of patients with associated medical problems at last follow-up was significantly reduced. Five patients (12.5%) underwent revisional surgery due to complications during follow-up. In conclusion, RBS in the elderly is associated with a reasonable complication rate and is effective in terms of weight loss and improvement of associated medical problems in a 5-year follow-up.

**Supplementary Information:**

The online version contains supplementary material available at 10.1007/s13304-024-01888-2.

## Introduction

Metabolic and Bariatric Surgery (MBS) is an effective treatment for patients with severe obesity, as it was shown to be associated with long-term constant weight loss, resolution of associated medical problems such as type 2 diabetes (T2D) and hypertension, decreased cancer rates and decreased mortality rates [1]. Due to its positive outcomes and the awareness of severe obesity implications, MBS has become more popular, and its rates are increasing worldwide [2].

Nowadays, Revisional Bariatric Surgery (RBS) compose 9–18% of all MBS [2–4]. The main indications for RBS are weight regain (WR) or insufficient weight loss (IWL)and 10–50% of patients with a previous restrictive procedure may require RBS [2, 5]. The main cause WR or IWL can vary they may be related to technical factors of the procedure performed but in general, are more commonly encountered in patients undergoing restrictive bariatric procedures in their background [6, 7].

MBS in the elderly patients is a controversial topic and in the new published guidelines for MBS [8], it is reported that there is no evidence for an age limit for MBS and elderly patients may require additional assessment including frailty. Many studies have been published throughout the last decade in elderly patients showing relatively good outcomes of MBS in the short- and long-term [9–12].

It is not uncommon that elderly patients are followed up in the MBS clinic and are found to have IWL or WR after a previous bariatric procedure, especially since restrictive procedure’s prevalence was very common in the twentieth century.

The objective of this study is to show the outcomes of patients aged ≥ 65 years old undergoing RBS due to IWR or WR after restrictive bariatric procedures.

## Methods

This study is based on a prospectively maintained patient registry in a single–tertiary bariatric referral center. Patients aged at least 65 years old undergoing RBS between January 2012–May 2022 were included in the study and their perioperative and last follow-up data were captured: In total 40 patients were included in the study, the indication for surgery were solely due to IWL or WR after a previous restrictive procedure. Patients were compared according to their previous restrictive procedure: 23 patients (57.5%) after laparoscopic adjustable gastric band (LAGB), and 16 patients (40%) after sleeve gastrectomy (SG). One patient (2.5%) was after siliastic ring vertical gastroplasty (SRVG) and was not included in the analysis. We chose to perform this comparison as these are the most commonly encountered procedures leading to IWL and WR and we wanted to investigate the safety and outcomes of revisional surgery after these procedures.

All patients were evaluated by the multi-disciplinary team and the indications for surgery were in accordance with recommended guidelines to the corresponding time period. In addition, all patients are further evaluated and discussed by the MBS exceptions committee.

### Data extraction

Additional data that were extracted included patients’ age, gender, body mass index (BMI), smoking, associated medical problems data including T2D, glycosylated hemoglobin levels, hypertension, hyperlipidemia, gastroesophageal reflux disease (GERD), osteoarthritis, Metabolic Associated Fatty Liver Disease (MAFLD). The time interval between surgeries was recorded as well.

### Surgical procedures

All surgical procedures were performed by the same MBS team with a standardized approach.

In all procedures, adhesiolysis was performed as indicated. Conversion from LAGB was preceded by removal of the band unless previously performed. Conversion from SG was initiated with transection of the SG at the level of the crow’s foot, with trimming of the sleeve when indicated.

#### One anastomosis gastric bypass (OAGB)

OAGB was performed in 22 patients (55%). All procedures were performed in a laparoscopic approach. The angle of His exposed and cleared till exposure of the left crus. The lesser sac was entered at the level of the crow’s foot. A long and narrow gastric pouch was constructed along bougie by applying a single horizontal staple line, followed by serial applications of staple cartridges up to the Angle of His. A linear-stapled anastomosis was then created between the gastric pouch and the small bowel, at 180–200 cm from the ligament of Treitz and a manualy closure of the opening. Routine blue dye leak test is performed.

#### Sleeve gastrectomy (SG)

SG was performed in 8 patients. The procedure begins by dissecting the greater omentum off the greater curvature from 4 cm proximal to the pyloric sphincter until the angle of His. The stomach is then vertically transected along a 36–40 Fr bougie. Routine blue dye leak test is performed.

#### Roux-en-Y gastric bypass (RYGB)

RYGB was performed in nine patients. A short gastric pouch was performed with a linear stapler along a bougie for calibration. The bowel was transected at 50–100 cm distal to the ligament of Treitz, defining the length of the biliopancreatic limb. A gastrojejunal anastomosis is then performed with a linear stapler and manual suturing of opening. A jejuno-jejunal anastomoses is then performed, constructing a 120–150 cm alimentary limb with manual suturing of opening.

### Perioperative outcomes

Data regarding 30-day outcomes of patients was captured and included: surgical complications, including leaks, bleeding, obstruction, infected fluid collections, reoperations, readmissions, length of stay (LOS), and mortality. The Clavien–Dindo (CD) grading system was used for complication grading and complications graded ≥ 3 were included [13].

### Mid-term follow-up outcomes

We withdrew data on last follow-up time, BMI, total weight loss (TWL). Resolution of associated medical problems (T2D, hypertension and hyperlipidemia) was recorded in accordance with recommended guidelines [14–16]. T2D remission was defined as a Hba1c level of < 6.5% that persists for at least 3 months without medications, hypertension remission was defined as blood pressure < 140/90 mmHg with no use of antihypertensive medications, and hyperlipidemia remission was defined after discontinuation of medications during the follow-up.

### Ethical concerns

The study was approved by the Institutional Review Board and was performed in accordance with the ethical standards of the institutional and/ or national research committee and with the 1964 Declaration of Helsinki and its later amendments or comparable ethical standards. Informed consent was obtained from all individual participants included in the study.

### Statistical analysis

Dichotomous data were analyzed using the Chi-square test. Continuous data were analyzed using the students' *t*-test. Continuous data are expressed as mean values ± standard deviation. All *p* values were derived from 2-tailed tests, and they were considered significant when *p* value was < 0.05. All calculations were conducted using the IBM SPSS version. 27.

## Results

Forty patients with a history of RBS were included in the study—23 patients (57.5%) with a background of LAGB (s/p LAGB), 16 patients (40%) with a background of SG (s/p SG), and 1 patient after SRVG (2.5%) which was not included in the comparative analysis. The indication for surgery was mainly due to WR or IWL in all patients. The baseline characteristics of patients are shown in Table 1—the mean age of patients was 67.2 ± 2.8 years old (range 65–74) with no significant difference between groups. There was a female predominance in the entre cohort of 24 women (61.5%). The mean BMI of the entire cohort was 38.3 ± 7.4 kg/m^2^ and was comparable between groups. There was no significant difference in associated medical problems including T2D, hypertension, hyperlipidemia, OSA, osteoarthritis, and MAFLD. There was a significantly higher rate of patients with GERD in patients s/p SG (68% vs 13%; *p* < 0.001). The number of patients consuming oral antidiabetics and insulin was 13 and 2 with no significant difference between groups (30.4% and 37.5%; *p* = 0.65, 4.3% and 6.2%; *p* = 0.79, respectively.

**Table 1:** Demographic features of elderly patients undergoing revisional BMS comparing previous LAGB to previous SG

	Total (*n* = 49)	s/p LAGB (*n* = 23)	s/p SG (*n* = 16)	*p* value
Age, mean ± SD, year	67.2 ± 2.8	67.9 ± 2.9	66.1 ± 2.3	0.051
Women, n (%)	24 (49%)	13 (56.5%)	11 (68.7%)	0.45
BMI, mean ± SD, kg/m^2^	38.3 ± 7.4	39.8 ± 4.2	36.2 ± 10	0.14
Smoking, n (%)	3 (6.1%)	2 (8.7%)	1 (6.2%)	0.78
T2D, n (%)	22 (44.9%)	13 (56.5%)	9 (56.2%)	0.98
%Hba1c, mean ± SD	6.9 ± 1.1	6.8 ± 1.2	7.1 ± 0.9	0.64
HTN, n (%)	26 (53%)	14 (60.9%)	12 (75%)	0.37
HL, n (%)	22 (44.9%)	11 (47.8%)	11 (68.7%)	0.2
GERD, n (%)	14 (28.6%)	3 (13%)	11 (68%)	< 0.001
OSA, n (%)	6 (12.2%)	3 (13%)	3 (18.7%)	0.63
Osteoarthritis, n (%)	5 (10.2%)	2 (8.7%)	3 (18.7%)	0.32
MAFLD, n (%)	21 (42.8%)	13 (56.5%)	8 (50%)	0.7
Anti-coagulant/aggregant use, n (%)	16 (32.7%)	11 (47.8%)	5 (31.2%)	0.39
*Surgical procedures*				
OAGB, n (%)	22 (44.9%)	(56.5%)13	9 (56.2%)	
SG, n (%)	8 (16.3%)	(34.7%)8	0 (0%)	
RYGB, n (%)	9 (18.4%)	(8.7%)2	7 (43.7%)	

*BMS* bariatric and metabolic surgery; *LAGB* laparoscopic adjustable gastric band; *SG* sleeve gastrectomy; *SD* standard deviation; *BMI* body mass index; *T2D* type 2 diabetes; *HTN* hypertension; *HL* hyperlipidemia; *GERD* gastroesophageal reflux disease; *OSA* obstructive sleep apnea; *MAFLD* metabolic associated fatty liver disease; *OAGB* one anastomosis gastric bypass; *RYGB* Roux-en-Y gastric bypass

The mean time interval between surgeries was 8.7 ± 5.1 years and was comparable between groups. The types of MBS are shown in Table 1 and include OAGB (*n* = 22), SG (*n* = 8) and RYGB (*n* = 9).

The postoperative (30-day) surgical complications are shown in Table 2—The rate of leaks, bleeding, obstruction and fluid collections was comparable between groups. There were three patients graded CD ≥ 3 with no significant difference between groups. The rate of 30-day readmission was significantly higher in patients s/p SG (18.7% vs 0%; *p* = 0.03).

**Table 2:** Postoperative (30-day) surgical complications of elderly patients undergoing RBS comparing previous LAGB to previous SG

	LAGB (*n* = 23)	SG (*n* = 16)	*p* value
Surgical complications, n (%)	2 (8.7%)	2 (12.5%)	0.70
Leaks, n (%)	1 (4.3%)	1 (6.2%)	0.79
Bleeding, n (%)	0 (0%)	1 (6.2%)	0.23
Obstruction, n (%)	0 (0%)	1 (6.2%)	0.23
Fluid collection/ Hematoma, n (%)	1 (4.3%)	0 (0%)	0.41
Reoperation, n (%)	0 (0%)	2 (12.5%)	0.08
Clavien–Dindo ≥ 3, n (%)	1 (4.3%)	2 (12.5%)	0.36
Mortality, n (%)	0 (0%)	1 (6.2%)	0.23
LOS, days, mean ± SD	4.5 ± 4.1	6.7 ± 4.1	0.11
30-day readmission, n (%)	0 (0%)	3 (18.7%)	0.03

*RBS* revisional bariatric surgery; *LAGB* laparoscopic adjustable gastric band; *SG* sleeve gastrectomy; *SD* standard deviation; *LOS* length of stay

There were two patients who required surgical revision due to major complications—The first patient is a 65-year-old male who underwent a conversion of SG to RYGB and due to hemorrhagic shock on postoperative day 1 underwent a laparoscopic exploration that revealed a large amount of blood and clots in the abdomen which eventually required a conversion to laparotomy which reveled oozing from the anastomotic staple line, the bleeding was oversewn and ceased. The patient recovered well and was discharged on postoperative day 6. The second patient is a 68-year-old female who underwent a conversion from SG to OAGB with an uneventful postoperative period, 2 weeks later she was readmitted due to septic shock with a diagnosis of anastomotic leak which required emergent laparotomy, lavage, drainage and the patient eventually died due to multi-organ failure.

We performed a comparative analysis regarding the type of RBS performed (See supplementary Table 1) and we found no significant difference in surgical complications, reoperations, readmissions and mortality. The LOS was significantly higher in patients undergoing RYGB when comparted to SG and OAGB (8.5 days vs 3.7 days and 4.7 days, respectively; *p* = 0.03).

**Fig. 1:**
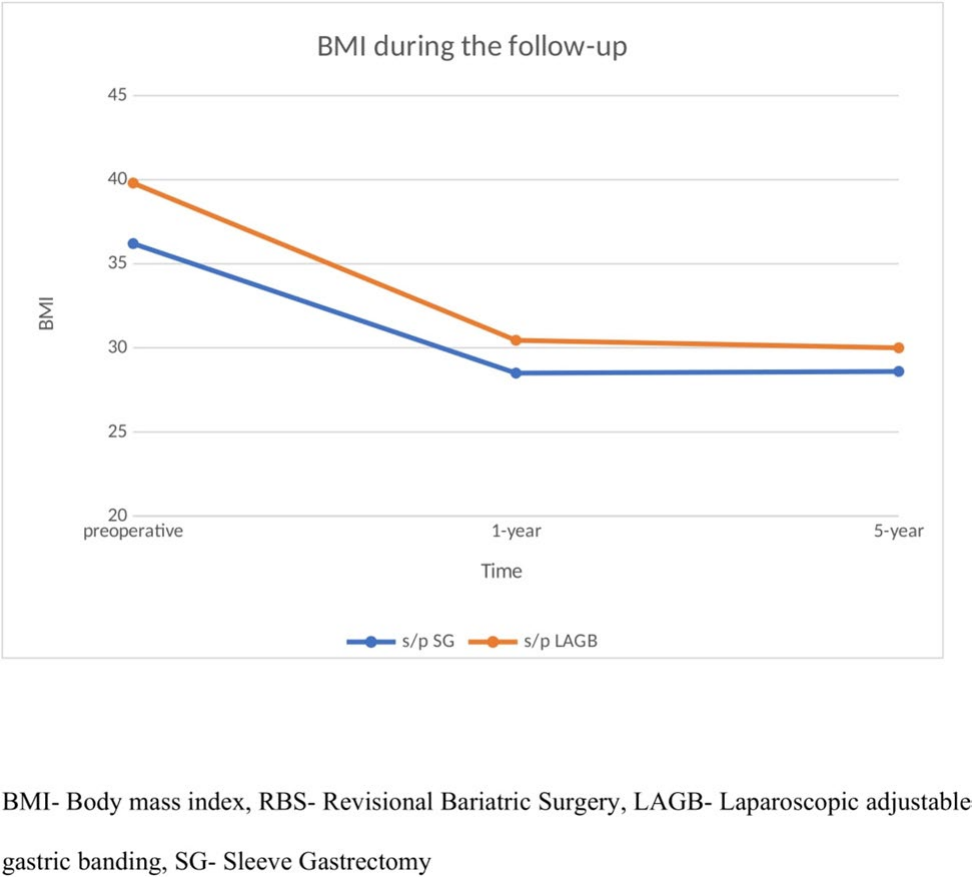
BMI trends during the follow-up in elderly patients undergoing RBS

Thirty-six patients were available to mid-term follow-up (10% loss to follow-up). The mean follow-up time of the cohort was 59.8 ± 30.6 months. The mid-term outcomes of patients are shown in Table 3—the mean BMI and TWL at last follow-up was 29.2 kg/m^2^ and 20.3%, respectively with no significant difference between groups. The rate of patients with obesity associated medical problems at last follow-up was significantly reduced and comparable between groups. The number of patients consuming oral antidiabetics in the last follow-up was 10 with no significant difference between groups (23% vs 25%; *p* = 0.9) and only one patient was still treated with insulin. The BMI trends of both groups are shown in Fig. 1. In regards to poor clinical response, 12 patients (≈ 30%) did not reach the TWL of at least 20%.

**Table 3:** Mid-term outcomes of elderly patients undergoing RBS comparing previous LAGB to previous SG

	s/p LAGB (*n* = 23)	s/p SG (*n* = 16)	*p* value
BMI at last follow-up, kg/m^2^, mean ± SD	29.9 ± 5.2	28.6 ± 6.3	0.50
%TWL at last follow-up, mean ± SD	24.1 ± 11.6	16.5 ± 12.4	0.08
T2D at last follow-up, n (%)	6 (28.5%)	4 (26.6%)	0.94
HTN at last follow-up, n (%)*	11 (52.4%)	3 (20%)	0.07
HL at last follow-up, n (%)	7 (33.3%)	2 (13.3%)	0.20

*RBS* revisional bariatric surgery; *OAGB* one anastomosis gastric bypass; *SG* sleeve gastrectomy; *BMI* body mass index; *SD* standard deviation; *EWL* excess weight loss; *TWL* total weight loss; *SD* standard deviation; *T2D* type 2 diabetes; *HTN* Hypertension; *HL* Hyperlipidemia

*All values calculated from number of patients available to follow-up

We performed a comparative analysis for weight loss outcomes and severe obesity associated medical problems at last follow-up in regards to the RBS performed. This analysis can be seen in Supplementary Table 2—OAGB was shown to have significantly lower BMI (27.8 kg/m^2^) and higher TWL (26%) when compared to SG (34.4 kg/m^2^, 11.8%, respectively) and RYGB (29.1 kg/m^2^, 16.7%, respectively) (*p* < 0.05). There was no significant difference in rates of T2D, hypertension and hyperlipidemia at last follow-up.

Additional revisional surgery due to complications during the follow-up was required in 5 patients (12.5%). One patient was converted from SG to OAGB due to sleeve stenosis, two patients after OAGB underwent laparoscopic exploration with omental patch due to marginal ulcer perforation, of which one required an additional revision due to small-bowel obstruction due to internal hernia which required reduction of hernia content and closure of the mesenteric defect and one patient had an OAGB anastomotic stricture which was treated with endoscopic dilatation which was complicated with a perforation that required surgical exploration and closure of the perforation.

Additional surgeries during the follow-up were required in two patients which included coronary artery bypass grafting (*n* = 1), and cataract surgery (*n* = 1).

In regards to malnutrition, one patient after RYGB had chronic diarrhea and severe iron deficiency which required pancreatic enzyme supplements and iron infusion with significant improvement of symptoms.

## Discussion

Restrictive bariatric procedures have dominated in the past 2 decades. Technical simplicity and good short- and medium-term outcomes have made both LAGB and SG the procedure of choice for many MBS surgeons. But simplicity has its price- with no malabsorptive component, and as longer follow-up accumulated, a significant sub-group of patients suffered from WR and required revisional surgery. Moreover, since WR usually peeks after 5–10 years from the index operation, the patients presenting with weigh regain are older, some of them over 65 years of age.

When considering re-operating a patient, age is a dominant factor. Revisional procedures are known to be of higher risk, with more complications expected. They also tend to be less effective, sometimes showing disappointing results. These drawbacks are highlighted when the surgical candidate is older, usually with significant co-morbidities.

Our study suggests that RBS is associated with a reasonable complication rate in the elderly population. We had only three patients with significant complications (CD > = 3) (7.7%), which is similar to the complication rates reported in previous reports on revisional bariatric surgeries on younger candidates [17]. Although patients s/p SG undergoing revisional surgery had a 12.5% major complication rate compared to 4.3% in s/p LAGB, this did not reach statistical significance due to the small sample size, in addition it is difficult to draw clear conclusions from this relatively small cohort. We did have a single mortality, which in such a small cohort account for a 2.6% mortality rate, but the impact of a single case in such small numbers makes it difficult to draw conclusions from this. It should be raised that elderly patients might need additional measures prior to RBS. In our opinion, age by itself should not define the patients’ physiologic and clinical status. However, it is naturally more common to encounter elderly patients with significant chronic medical conditions and frailty which might require a more careful, evaluation, optimization of chronic medical diseases, and careful patient selection.

It seems there is a trend toward a more complicated course following a SG compared to LAGB– in reoperations (2 vs 0 *p* = 0.08), LOS (6.7 days vs 4.5 days, *p* = 0.11) and 30-day readmission rates (3 vs 0, *p* = 0.03). This should not surprise us since the gastric band does not involve resection of the stomach therefore less adhesions are encountered. Also, 8 bands were converted to a sleeve gastrectomy and 15 to some form of bypass, whereas all sleeves were converted to a bypass procedure—which can be more technically challenging and are usually associated with higher complication rates [18].

We managed to collect mid-term follow-up data on 36 patients (92.3%). Our patients achieved an average weight loss of over 20% of their total body weight, and an average decrease of 9 BMI points at an average of 5 years follow-up. Although not statistically significant, LAGB revisions had nearly significant higher TWL outcomes compared to SG revisions (TWL 24.1% vs 16.5%; *p* = 0.08). This is similar to previous reports on revisions after LAGB and SG—with SG revisions trailing LAGB revisions regarding weight loss outcomes [17, 19]. TWL < 20% was considered a poor clinical response and this was seen in 30% of patients which is reasonable when considering it from the revision date and in our opinion is only one of the markers determining the outcome postoperatively.

We also observed good remission rates in associated medical problems—T2D, HTN, and HL. Some would argue that these medical conditions in the elderly, being long-lasting and of more chronic nature, would be more resilient even after significant weight loss—but it is clear that this is another advantage of revisional surgery in this patient population [20].

This is the largest cohort of revisional procedures in the elderly population (> 65 years old). Holtestaul et al. [10] reported their experience with revisional surgeries in patients above 65 years of age, with only 15 cases which were compared to 130 revisional procedures in younger candidates. They found similar outcomes regarding weight loss, morbidity, and mortality and concluded that revisional surgery is safe and effective in the elderly.

Another study by Zarzycki et al. [12] summarized 55 cases of revisional surgeries on patients 60 years or older. They found lower complication rates compared to younger patients undergoing revisions, but lower remission rates in obesity related co-morbidities.

It would be expected that the rate of nutritional complications will likely be higher as this population are more prone to nutritional complications especially after hypoabsorptive procedures (i.e., OAGB), however, there was only one patient from the entire cohort with chronic diarrhea and iron deficiency anemia after RYGB and no nutritional issues after OAGB. We assume that this is probably due to a small cohort of patients with a nearly 10% loss to follow-up which could include other patients.

When relating to the type of RBS performed we showed that OAGB had significantly higher TWL at last follow-up when compared to SG and RYGB. Despite this, we should emphasize again the small cohort size and a comparison of such small numbers can not draw clear conclusions. OAGB is gaining popularity in the last decade owing to its relatively short learning curve, safety and effectiveness [21]. Musella et al. [22] reported a multi-central retrospective anaylsis of patients undergoing OAGB after failed restrictive procedures and showed a 74.5% excess BMI loss at 20.8 months follow-up with 8.6% overall complication rate. In an expert’s modified Delphi consensus on the best approach for redo surgeries after SG, OAGB was agreed by 84.7% of the committee to be an acceptable procedure after SG [23].

Our study has several limitations which include a retrospectively designed study of non-randomized groups which can result in underreporting and selection bias. In addition, the cohort is relatively small (*n* = 49). We also report a diversity of two primary procedures—LAGB and SG—being converted into three different surgical procedures. There is also an 8% loss to follow-up which could skew the data. We could not determine resolution of other severe obesity associated medical problems (such as MAFLD) as only a small proportion of patients underwent an abdominal ultrasound during the follow-up.

Despite these limitations, to our knowledge this is the study with the largest number of elderly patients (> 65 years) undergoing RBS and there is a relatively long follow-up time (59.8 months).

## Conclusions

RBS in elderly patients over 65 years old is effective and with a reasonable complication rate. The cohort is relatively small with no control group and clear conclusions could not be drawn regarding safety. There was no significant difference between LAGB to SG in patients’ mid-term outcomes after revisional surgery. This patient’s population benefits from RBS with significant, long-lasting weight loss and improvement in obesity associated medical problems. OAGB as a RBS showed better TWL rates. Careful patient selection is mandatory to keep complication rate low and to ensure a true improvement in physical wellness and quality of life. Prospective, larger scale studies are necessary to further evaluate thus sub-group of patients as we are likely to encounter more of them with the increase in life expectancy.

## Supplementary Information

Below is the link to the electronic supplementary material.

Supplementary file1 (DOCX 16 KB)

Supplementary file2 (DOCX 15 KB)
