# Supplementary material for: Revision of restrictive bariatric procedures in elderly patients: results at a 5-year follow-up
Source: Updates Surg. 2024 May 26;76(8):2825–31. doi: 10.1007/s13304-024-01888-2 (PMC11628429; doi:10.1007/s13304-024-01888-2)
Supplement: Supplementary file 1 — Supplementary file1 (DOCX 16 KB) [file 13304_2024_1888_MOESM1_ESM.docx]

| P value | RYGB (n=9) | OAGB (n=22) | SG (n=8) |  |
| --- | --- | --- | --- | --- |
| <0.001 | 2 (22%) | 13 (59%) | 8 (100%) | Previous LAGB, n (%) |
| <0.001 | 7 (78%) | 9 (41%) | 0 (0%) | Previous SG, n (%) |
| 0.32 | 2 (22%) | 2 (9.1%) | 0 (0%) | Surgical complications, n (%) |
| 0.46 | 0 (0%) | 2 (9.1%) | 0 (0%) | Leaks, n (%) |
| 0.19 | 1 (11%) | 0 (0%) | 0 (0%) | Bleeding, n (%) |
| 0.14 | 0 (0%) | 0 (0%) | 1 (12.5%) | Obstruction, n (%) |
| 0.19 | 1 (11%) | 0 (0%) | 0 (0%) | Fluid collection/ Hematoma, n (%) |
| 0.59 | 1 (11%) | 1 (4.5%) | 0 (0%) | Reoperation, n (%) |
| 0.17 | 2 (22%) | 1 (4.5%) | 0 (0%) | Clavien Dindo ≥ 3, n (%) |
| 0.69 | 0 (0%) | 1 (4.5%) | 0 (0 %) | Mortality, n (%) |
| 0.03 | 8.5 ± 6.2 | 4.7 ± 3.5 | 3.7 ± 1.3 | LOS, days, mean ± SD |
| 0.66 | 1 (11%) | 2 (9.1%) | 0 (0%) | 30-day readmission, n (%) |

Supplementary Table 1

Postoperative (30-day) surgical complications of elderly patients undergoing RBS comparing different MBS types after LAGB and SG

MBS – Metabolic and Bariatric Surgery; RBS- Revisional Bariatric Surgery; LAGB – Laparoscopic adjustable gastric banding; SG – sleeve gastrectomy ;OAGB – One anastomosis gastric bypass; RYGB – Roux en Y gastric bypass SD= Standard Deviation; LOS = Length of Stay
