# Supplementary material for: Revision of restrictive bariatric procedures in elderly patients: results at a 5-year follow-up
Source: Updates Surg. 2024 May 26;76(8):2825–31. doi: 10.1007/s13304-024-01888-2 (PMC11628429; doi:10.1007/s13304-024-01888-2)
Supplement: Supplementary file 2 — Supplementary file2 (DOCX 15 KB) [file 13304_2024_1888_MOESM2_ESM.docx]

Supplementary Table 2

| P value | RYGB (n=9) | OAGB (n=20) | SG (n=7) |  |
| --- | --- | --- | --- | --- |
| 0.03 | 29.1 ± 3 | 27.8 ± 6.3 | 34.4 ± 4.3 | BMI at last follow-up, kg/m^2^, mean ± SD |
| 0.01 | 16.7 ± 12.3 | 26 ± 12.3 | 11.8 ± 6.9 | %TWL at last follow-up, mean ± SD |
| 0.48 | 3 (33.3%) | 4 (20%) | 3 (42.9%) | T2D at last follow-up, n (%) |
| 0.56 | 3 (20%) | 7 (35%) | 4 (57%) | HTN at last follow-up, n (%) * |
| 0.36 | 1 (11%) | 5 (25%) | 3 (42.9%) | HL at last follow-up, n (%) |

Mid-term outcomes of elderly patients undergoing RBS comparing MBS performed*

MBS- Metabolic and Bariatric Surgery RBS- Revisional Bariatric Surgery; OAGB = One Anastomosis Gastric Bypass; SG = sleeve gastrectomy; RYGB = Roux en Y gastric bypass BMI = Body Mass Index; SD = Standard Deviation; EWL = Excess weight loss; TWL = Total Weight Loss; SD= Standard Deviation; T2D = Type 2 Diabetes; HTN = Hypertension; HL = Hyperlipidemia

*All values calculated from number of patients available to follow-up
